# Supplementary material for: Sensitivity of nasopharyngeal, oropharyngeal, and nasal wash specimens for SARS-CoV-2 detection in the setting of sampling device shortage
Source: Eur J Clin Microbiol Infect Dis. 2020 Sep 17;40(2):441–5. doi: 10.1007/s10096-020-04039-8 (PMC7494432; doi:10.1007/s10096-020-04039-8)
Supplement: Supplementary file 1 — (DOCX 28 kb) [file 10096_2020_4039_MOESM1_ESM.docx]

**Supplementary data: tables**

|  | DMEM | | UTM | | ∆Ct values | |
| --- | --- | --- | --- | --- | --- | --- |
| Patient | ORF 1 | E-gene | ORF 1 | E-gene | ORF 1 | E-gene |
| a | ND | ND | 36.43 | ND | **NA** | **NA** |
| b | 28.8 | 29.39 | 29.03 | 30.08 | **0.23** | **0.69** |
| c | ND | ND | ND | ND | **NA** | **NA** |
| d | 31.17 | 32.55 | 28.89 | 29.96 | **2.81** | **2.59** |
| e | ND | 37.81 | 36.3 | 37.82 | **NA** | **0.01** |
| f | 24.95 | 25.43 | 25.01 | 25.11 | **0.06** | **0.32** |
| g | 20.87 | 21.6 | 20.84 | 20.94 | **0.03** | **0.66** |
| h | 32.22 | 33.64 | 31.81 | 32.73 | **0.41** | **0.91** |
| i | 28.8 | 29.51 | 28.9 | 29.41 | **0.1** | **0.1** |
| j | 29.25 | 30.14 | 29.17 | 30.04 | **0.08** | **0.1** |

**Supplementary table 1a. Comparison of DMEM and UTM media.**

The delta Ct value for target ORF 1 between the two transport media containing the same specimen ranged between 0.01 and 2.59. One specimen turned out negative in both media. Two specimens were very close to the detection limit and yielded undetectable results for one or more targets (patient a: ND/ND and 36.43/ND, patient e: ND/37.81 and 36.3/37.82). ND: not detected. NA: not applicable.

| Transport media | Sample Dilution | ORF1 target | E-gene target | Delta CT ORF1 target | Deta CT E-gene target |
| --- | --- | --- | --- | --- | --- |
| UTM | 0 | 33.38 | 33.77 |  |  |
| DMEM | 0 | 33.18 | 33.94 | -0.2 | 0.17 |
| UTM | 10-1 | 35.73 | 37.07 |  |  |
| DMEM | 10-1 | 35.45 | 36.05 | -0.28 | -1.02 |
| UTM | 10-2 | 37.87 | neg |  |  |
| DMEM | 10-2 | 37.66 | neg | -0.21 | ND |
| UTM | 10-3 | neg | neg |  |  |
| DMEM | 10-3 | neg | neg | ND | ND |
| UTM | 10-4 | neg | neg |  |  |
| DMEM | 10-4 | neg | neg | ND | ND |

**Supplementary table 1b. Comparison of DMEM and UTM media for detection of low SARS-CoV-2 viral loads.**

Five additional samples with high Ct values were added to compare the DMEM and the UTM in low viral loads specimens. The delta Ct values seemed comparable to those obtained with lower Ct values in figure 1a.

| a. | NP swab + 1ml DMEM + 2ml NaCl | | 2ml NASAL WASH + 1ml DMEM | | delta CT | |
| --- | --- | --- | --- | --- | --- | --- |
| patients | ORF1 Ct value | E-gene Ct value | ORF1 Ct value | E-gene Ct value | ORF1 | E-gene |
| 1 | 23.5 | 23.9 | 30.56 | 31.21 | 7.06 | 7.31 |
| 2 | 34.28 | 35.33 | 27.78 | 28.67 | -6.5 | -6.66 |
| 3 | 23.15 | 23.63 | 30.88 | 31.88 | 7.73 | 8.25 |
| 4 | 30.95 | 32.84 | 32.67 | 33.95 | 1.72 | 1.11 |
| 5 | 24.03 | 24.06 | 30.97 | 31.51 | 6.94 | 7.45 |
| 6 | 23.94 | 24.32 | 29.88 | 30.93 | 5.94 | 6.61 |
| 7 | 30.38 | 31.44 | 31.11 | 31.71 | 0.73 | 0.27 |
| 8 | 19.11 | 19.43 | 24.34 | 24.65 | 5.23 | 5.22 |
| 9 | 29.54 | 30.64 | 31.21 | 32.34 | 1.67 | 1.7 |
| 10 | 15.45 | 15.71 | 16.54 | 16.64 | 1.09 | 0.93 |
| 11 | 31.1 | 32.28 | 33.86 | 35.5 | 2.76 | 3.22 |
| 12 | 31.71 | 33.79 | 31.83 | 32.8 | 0.12 | -0.99 |
| 13 | 33.05 | 34.48 | 26.23 | 26.69 | -6.82 | -7.79 |
| 14 | 16.53 | 16.69 | 15.08 | 15.32 | -1.45 | -1.37 |
| 15 | 33.46 | 35.12 | NEG | NEG | NA | NA |
| 16 | 26.17 | 26.71 | 24.42 | 24.47 | -1.75 | -2.24 |
| 17 | 17.57 | 18.01 | 19.83 | 20.12 | 2.26 | 2.11 |
| 18 | 23.09 | 23.9 | 26.63 | 28.32 | 3.54 | 4.42 |
| 19 | 30.81 | 32.7 | 34.62 | 37.18 | 3.81 | 4.48 |
| 20 | 33.9 | 35.96 | 33.38 | 34.81 | -0.52 | -1.15 |
| Mean delta CT |  | | | | 1.76631579 | 1.73052632 |
| b. | **NP swab + 3ml DMEM** | | **OP swab + 3ml DMEM** | | **Delta Ct values** | |
| patients | ORF1 Ct value | E-gene Ct value | ORF1 Ct value | E-gene Ct value | ORF1 | E-gene |
| 21 | NEG | NEG | NEG | NEG | NA | NA |
| 22 | 28.4 | 29 | 34.2 | 36.6 | 5.8 | 7.6 |
| 23 | 21.1 | 22 | 26.88 | 27.67 | 5.78 | 5.67 |
| 24 | 31.2 | 32.47 | 35.43 | 37.83 | 4.23 | 5.36 |
| 25 | 25.4 | 26.3 | 29.4 | 30.3 | 4 | 4 |
| 26 | 14.7 | 15.8 | 18.3 | 18.7 | 3.6 | 2.9 |
| 27 | 27.09 | 27.5 | 30.28 | 30.73 | 3.19 | 3.23 |
| 28 | 18 | 18.38 | 20.71 | 21.14 | 2.71 | 2.76 |
| 29 | 20.69 | 21.34 | 23.33 | 23.56 | 2.64 | 2.22 |
| 30 | 20.9 | 21.6 | 23.4 | 23.9 | 2.5 | 2.3 |
| 31 | 31.95 | 34.17 | 34.4 | 36.9 | 2.45 | 2.73 |
| 32 | 23.08 | 23.61 | 25.33 | 25.72 | 2.25 | 2.11 |
| 33 | 30.75 | 32 | 32.99 | 33.85 | 2.24 | 1.85 |
| 34 | 29.25 | 30.13 | 31.36 | 32.62 | 2.11 | 2.49 |
| 35 | 22.78 | 23.45 | 24.79 | 25.24 | 2.01 | 1.79 |
| 36 | 31.6 | 32.5 | 33 | 34 | 1.4 | 1.5 |
| 37 | 29.03 | 29.97 | 29.88 | 30.69 | 0.85 | 0.72 |
| 38 | 34.16 | 36.8 | 34.49 | 35.96 | 0.33 | -0.84 |
| 39 | 30.13 | 31.57 | 30.45 | 31.28 | 0.32 | -0.29 |
| 40 | 31.5 | 34 | 31.7 | 33.6 | 0.2 | -0.4 |
| 41 | 32.9 | 35.1 | 32.9 | 34.8 | 0 | -0.3 |
| 42 | 33.69 | 36.5 | 33.12 | 34.43 | -0.57 | -2.07 |
| 43 | 22.5 | 23.00 | 21.5 | 22.30 | -1 | -0.7 |
| 44 | 32.1 | 33.56 | 30.91 | 32.96 | -1.19 | -0.6 |
| 45 | 30.26 | 31.01 | 27.53 | 28.13 | -2.73 | -2.88 |
| 46 | 34.1 | 36.6 | 30.6 | 32.3 | -3.5 | -4.3 |
| 47 | 29.03 | 30.05 | 24.79 | 25.42 | -4.24 | -4.63 |
| 48 | NEG | NEG | NEG | NEG | NA | NA |
| 49 | 34.84 | 36.83 | 33.14 | 35.2 | -1.7 | -1.63 |
| Mean delta CT |  | | | | 1.24740741 | 1.13296296 |

**Table 2. Sampling methods comparison.**

**a**. Ct values of NP swab specimens compared to Ct values of NW specimens by RT-PCR in 20 patients. The mean delta Ct value shows a slightly better overall sensitivity with NP swabs (average 1.7). Patient 15’s NW sample was negative despite a positive NP specimen.

**b.** Ct values of NP swab specimens compared to Ct Values of OP swab specimens in RT-PCR in 29 patients. Patients 1 and 28 were negative both in NP and OP swabs. The mean delta Ct value shows a slightly overall better sensitivity with NP swabs (average 1.2-1.3). The specimen collection was done at the same time.
